# The Role of Hepatitis C Virus Core Antigen Testing in the Era of Direct Acting Antiviral Therapies: What We Can Learn from the Protease Inhibitors

**DOI:** 10.1371/journal.pone.0163900

**Published:** 2016-10-06

**Authors:** Linh Thuy Nguyen, Emma Gray, Aisling O'Leary, Michael Carr, Cillian F. De Gascun, Irish Hepatitis C Outcomes Research Network

**Affiliations:** 1 National Virus Reference Laboratory, University College Dublin, Dublin, Ireland; 2 School of Medicine and Medical Science, University College Dublin, Dublin, Ireland; 3 Ireland Vietnam Blood-Borne Virus Initiative (IVVI), Dublin, Ireland and Hanoi, Vietnam; 4 National Centre for Pharmacoeconomics in Ireland, St James’s Hospital, Dublin, Ireland; Temple University School of Medicine, UNITED STATES

## Abstract

Direct-acting antiviral (DAA) therapies have revolutionised the treatment of hepatitis C virus (HCV). The financial cost of DAAs however is significant, and first generation protease inhibitors (PIs) also require frequent monitoring of viral RNA levels to guide treatment. In this context, we examined the relevance of HCV antigen testing to evaluate the potential role in monitoring virological response to HCV antiviral treatment with the PI-based triple therapies, telaprevir (TVR) and boceprevir (BOC). Chronic HCV-infected individuals (n = 152) enrolled in the Irish Hepatitis C Outcomes Research Network (ICORN) study were prospectively analysed for baseline markers and the early viral kinetics associated with SVR. The sustained virological response (SVR) rates in the cohort receiving TVR and BOC were 87.3% and 73.8%, respectively. Baseline factors associated with successful outcome in TVR therapy were age (*P* = 0.0098), *IFNL3* genotype (*P* = 0.0330) and viral load (*P* = 0.0456). RNA level at week 4 (*P* = 0.0068) and viral antigen negativity at week 2 (*P* = 0.0359) were predictive of SVR for TVR-based therapy. In BOC therapy, prior interferon treatment (*P* = 0.0209) and *IFNL3* genotype (*P* = 0.0410) were baseline predictors of SVR. Evidence of viraemia based either on viral RNA or antigen at week 4 predicted SVR in these patients. Our data showed that rapid decline of HCV antigen to negative level at week 2 in TVR treatment and <0.96 log fmol/l in BOC treatment after commencement of PI triple therapy were associated with SVR. HCV antigen measurement should be considered as a potential alternative for monitoring treatment response during DAA-based regimens.

## Introduction

Direct-acting antivirals (DAAs) have transformed the treatment of hepatitis C virus (HCV) infection, resulting in higher rates of sustained virological response (SVR) [1]. However, the high costs and the significant toxicities associated with first generation regimens that combine a protease inhibitor (PI) with pegylated interferon-α and ribavirin (PEG-IFN/RBV) [1] stimulated interest in the identification of individual predictors of patient response in clinical development programmes. This led to the proposal of decision rules to determine whether treatment should be abandoned (futility rules), abbreviated (response-guided therapy) or whether patients should complete the full treatment course [2]. This decision-making process requires frequent viral RNA testing to facilitate adherence to licensed recommendations for PI-based triple therapies.

Despite the high specificity, sensitivity and reproducibility of HCV RNA quantification assays, their costs can constrain their utility in resource-limited settings. Furthermore, molecular assays—typically performed in batches—may preclude sufficiently short turn-around times to facilitate efficient HCV DAA treatment decisions in the clinic [3]. Conversely, the CE-marked HCV core antigen quantification assay (ARCHITECT HCV Ag assay, Abbott Diagnostics) [4] constitutes a rapid, more economical and easier-to-perform method, with good correlation to HCV RNA assays [5–7]. These advantages were previously investigated to assess the ability of antigen testing to supplement molecular viral load testing for monitoring treatment of PEG-IFN/RBV therapy. The results showed the potential clinical utility of HCV core antigen (HCVcAg) at early stages in dual PEG-IFN/RBV therapy to predict treatment response as early as day 3 [8], week 1 [7, 9] or week 2 [3, 9–11]. The present study aims to determine the utility of HCVcAg testing in the era of DAA-based triple therapy.

## Materials and Methods

### Patient population

Plasma/serum specimens obtained from chronically HCV-infected (CHC) genotype 1-infected patients (n = 152) receiving either telaprevir (TVR) (n = 110) or boceprevir (BOC) (n = 42) in triple therapy with PEG-IFN/RBV were received from seven tertiary care hospitals. Ethical approval for the ICORN study was obtained from the St. James’/Tallaght Hospital Research Ethics Committee (2012/47/08 RTC) in December 2012. Participants were enrolled in the study following the provision of written informed consent.

### Methods

HCV RNA measurements (viral loads) were quantified using the Abbott Molecular m2000 RealTime System (Abbott Molecular Laboratories, Wiesbaden, Germany) prior to initiation of treatment (baseline), and on-treatment at weeks 4, 8, 12, 24 and 48 and 24 weeks post-treatment cessation for evaluation of virological response following guidelines. For analysis of early viral antigen kinetics, HCVcAg levels were examined with the ARCHITECT HCV Ag (Abbott Diagnostics, Wiesbaden, Germany) assay at the following time-points: baseline (n = 100), week 1 (n = 35), week 2 (n = 45) and week 4 (n = 103) for samples obtained from TVR-treated patients; and baseline (n = 39) and week 4 (n = 37) for samples from BOC-treated patients. HCVcAg levels (log_10_ fmol/l) were correlated with HCV viral loads (log_10_ IU/ml) and treatment outcomes to determine predictive values. To include all data for analysis, detectable RNA levels below the limits of quantification of the viral load assay (12 IU/ml) were represented as 6 IU/ml to obtain the log transformed value of 0.78 log_10_ IU/ml. RNA not detected was assigned with the log_10_ value of 0. HCVcAg levels <3 fmol/l (interpreted as non-reactive by the manufacturer) were assigned with a log_10_ value of 0.

*IFNL3* rs12979860 single nucleotide polymorphism (SNP) analysis was performed by allelic discrimination real-time PCR as described previously [12]. HCV genotyping was performed by Innogenetics VERSANT HCV genotype 2.0 (Siemens Healthcare, Milan, Italy) or the RealTime HCV Genotype II (Abbott Molecular Laboratories, Wiesbaden, Germany) assays. Identification of viral subtype based on *NS3* sequencing and DRMs in the NS3 protease were performed as previously described [13]. Human interferon-ɣ inducible protein 10 (IP-10) was quantified by using the Quantikine human CXCL10/IP-10 immunoassay (R&D Systems). Human serum microRNAs (miRs), including miR-122 and let-7b, were measured by relative quantification real-time PCR using the TaqMan Small RNA assays on the ABI7500 Fast Real-time PCR system as described [14].

### Definitions

The definitions of the European Association for the Study of the Liver (EASL) treatment management algorithm were followed [15]. SVR was defined as an undetectable HCV RNA level 24 weeks after treatment cessation. Rapid virological response and early virological response were were referred as RNA negativity at week 4 and week 12, respectively after commencement of TVR-based therapy. Early response and late response were referred as RNA negativity at week 8 and week 12, respectively for BOC-based therapy. HCV RNA reduction was referred to as a log_10_ IU/ml decrease of HCV VL and HCVcAg reduction was defined as a log_10_ fmol/l decrease of HCVcAg level in plasma/sera between baseline and subsequent time points for each assay.

### Statistical analysis

The statistical significance of differences between groups was analysed by Fisher’s exact test for categorical variables and Mann-Whitney U-test for continuous variables. Correlation between levels of HCVcAg and RNA was measured by Spearman’s rank correlation coefficient tests. Optimal predictive values were assessed by calculating the areas under the receiver operating characteristics curve (AUC value). Sensitivity (SEN), specificity (SPE), positive predictive value (PPV), negative predictive value (NPV) and accuracy (ACC) were calculated to determine the reliability of predictors of the response to each therapy. Each cut-off value for continuous variables was decided by the Youden index method on the basis of the receiver operating characteristics curve (ROC), as the inflection point at which the sum of sensitivity and (specificity-1) was the maximum. Two-tailed *P* values less than 0.05 were considered statistically significant. The statistical analysis was performed with software MedCalc v14.8.1.

## Results

### Baseline characteristics of the study cohort

In total, 72.4% (110/152) of the study cohort were treated with TVR and 27.6% (42/152) received BOC. The median ages were 45 years in the TVR and 48 years in the BOC group with a male predominance (67.3% TVR; 70.7% BOC). The proportions of patients with cirrhosis were 23.1% (TVR) and 34.1% (BOC). 39.1% of TVR-treated patients and 26.2% of BOC-treated patients were treatment-experienced individuals who did not achieve an SVR with previous PEG-IFN/RBV regimen. The major homozygous *IFNL3* rs12979860 CC frequency was 32.7% in TVR-treated patients and 23.8% in BOC-treated patients respectively. The majority of the study cohort was chronically infected with HCV GT1a: 67.3% in the TVR group and 70.0% in the BOC group; the remainder was infected with HCV GT1b. As previously reported, the naturally occurring RAMs conferring resistance to PIs, T54S, V55A and I132V, were detected in 33.3% of TVR-receiving patients; and V36L and V55A were detected in 6.5% of BOC-receiving patients [13].

With regards to baseline characteristics, we determined a statistically significant association of both older age and *IFNL3* CC genotype with cirrhosis in the cohort (Table 1). The group with cirrhosis (n = 39; median age, 51 years; range, 32–70) was significantly older than the group without cirrhosis (n = 110; median age, 44 years; range, 19–68; *P* = 0.0005). The proportion of individuals who developed cirrhosis in the *IFNL3* CC group (41.3%) was significantly higher than the non-CC group (19.4%, *P* = 0.008).

**Table 1:** Baseline factors and their association with cirrhosis status in the study cohort.

	Cirrhosis(N = 39)	No cirrhosis(N = 110)	*P*-value
Age (years), median (range)	51 (32–70)	44 (19–68)	**0.0005**
rs12979860 CC, %	48.7%	24.5%	**0.0080**
Male, %	74.4%	71.8%	0.8368
Treatment experienced, %	33.3%	35.5%	0.8476
HCV GT1a, %	65.8%	68.0%	0.8404

### Therapeutic efficacy

In the TVR group, 87.3% (96/110) achieved SVR; 9.1% (10/110) were non-responders and 3.6% (4/110) relapsed after completing the treatment. In the BOC group, 73.8% (31/42) achieved SVR; 16.7% (7/42) were non-responders and 9.5% (4/42) were relapsers (Table 2). Due to the differing therapies and rates of outcomes observed between the two groups, the subsequent analysis was performed separately for each drug.

**Table 2:** Treatment efficacy of the cohort receiving TVR, BOC or both PIs.

	TVR(N = 110)	BOC(N = 42)	All(N = 152)
Responder	87.3% (96/110)	73.8% (31/42)	83.6% (127/152)
Non-responder	9.1% (10/110)	16.7% (7/42)	11.2% (17/152)
Relapse	3.6% (4/110)	9.5% (4/42)	5.3% (8/152)

### Pre-treatment factors contributing to SVR

**Fig 1:**
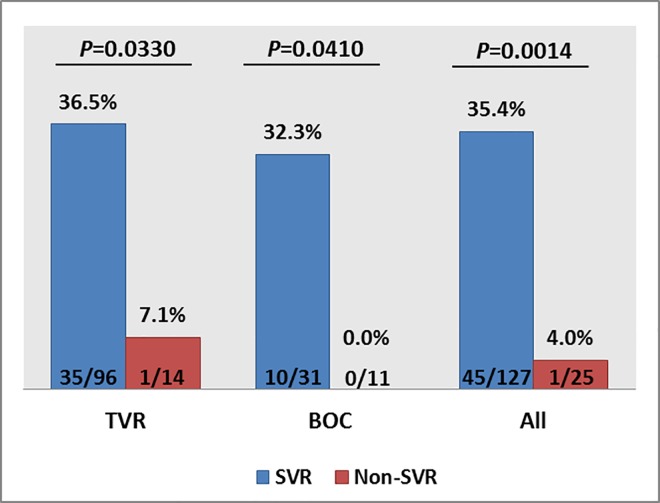
Frequencies of the rs12979860 CC genotype for PI therapies and association with treatment outcome.

The results of univariate analysis of pre-treatment factors contributing to SVR are presented in Table 3. Significantly, for TVR, younger age was associated with SVR (*P* = 0.0098) and *IFNL3* CC carriers were more likely to have SVR (*P* = 0.0330). In the BOC cohort, treatment-naïve individuals had a higher likelihood of achieving SVR than treatment-experienced individuals (*P* = 0.0209). As with the TVR cohort, BOC-treated patients of *IFNL3* CC genotype were also more likely to achieve a higher SVR rate (*P* = 0.041). In separated analysis for each drug, there was a lower statistical association of *IFNL3* rs12979860 genotypes and outcome; however, when the two drugs are combined for analysis, *IFNL3* rs12979860 CC was significantly associated with SVR (*P* = 0.0014) (Fig 1).

**Table 3:** Baseline characteristics of the PI-treated cohorts and the association of pre-treatment factors with SVR.

Characteristics	TVR treatment (N = 110)			BOC treatment (N = 42)		
	SVR(N = 96)	No SVR(N = 14)	*P*-value	SVR(N = 31)	No SVR(N = 11)	*P*-value
Age (years), median(range)	44(26–70)	52(44–67)	**0.0098**	48.0(19–69)	48.0(35–68)	0.9772
Male, %	74	71.4	1.0000	67.7	80.0	0.6937
Treatment naïve, %	63.5	42.9	0.1540	83.9	45.5	**0.0209**
Cirrhosis, %	21.1	38.5	0.1736	32.3	40.0	0.7116
rs12979860 CC, %	36.5	7.1	**0.0330**	32.3	0.0	**0.0410**
IP-10 (pg/ml), median(range)	353.7(95.9–2328.5)	271.8(151.5–787.7)	0.9612	308.4(91–1238.6)	499.1(125.7–1016.6)	0.1158
miR-122 (Ct), median(range)	31.09(27.6–35.2)	30.89(29.0–34.3)	0.6612	30.6(26.6–34.2)	30.9(29.1–32.5)	0.7997
let-7b (Ct), median(range)	30.68(27.3–36.1)	30.85(28.7–33.0)	0.5188	30.4(26.6–33.6)	31.0(30.2–32.5)	0.3101
HCV GT1a, %	64.8	84.6	0.2119	62.1	90.9	0.1244
HCV RAMs, %	36.1	16.7	0.3212	4.8	10.0	1.000

### On treatment factors contributing to SVR

**Fig 2:**
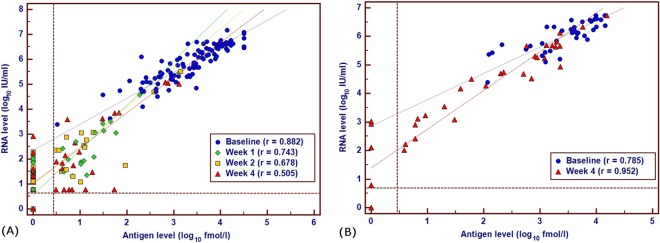
**Scatter plots depicting the correlation between HCV RNA (log**_**10**_ **IU/ml) and HCVcAg (log**_**10**_ **fmol/l) measurements in samples from the cohorts receiving TVR (A) and BOC (B) at early time points.** The lower left hand corner restricted by the dashed lines indicates the area where both RNA and antigen are not detected by the respective assays.

Prior to investigating the clinical utility of early viral kinetics based on viral RNA and HCVcAg, the analytical performance of these viral markers was evaluated. For TVR therapy, a good correlation (r = 0.882) between viral load and antigen was observed at baseline (Fig 2A). However, the HCV RNA-HCVcAg correlation decreased at subsequent time points; this was due to the rapid decrease of HCVcAg below the limits of detection of the viral antigen assay (<3 fmol/l), whilst viral RNA remained detectable. This corresponded with a decreased concordance between detectable RNA and reactive HCVcAg after starting treatment: at baseline, the concordance of the positivity rates of the two viral markers is 100% (100/100); after one, two and four weeks, antigen reactive samples were 52.9% (18/34), 37.5% (15/40) and 29.6% (21/71), respectively of samples with detectable viral RNA. For BOC therapy (Fig 2B), there was a moderate correlation of HCV RNA and HCVcAg correlation at baseline (r = 0.785); however, the correlation increased at week 4 (r = 0.952). The concordance of the positivity rates for the two viral markers at baseline and four weeks post-treatment was 100% (39/39) and 80% (28/35), respectively.

In terms of clinical utility, the results of univariate analysis of on-treatment factors contributing to SVR are shown in Tables 4 and 5. For TVR therapy (Table 4), significant differences between the SVR and non-SVR groups were noted regarding the HCV RNA level at baseline (*P* = 0.0456), RNA levels at week 4 (*P* = 0.0068) and HCVcAg negativity at week 2 (*P* = 0.0359). For BOC therapy (Table 5), SVR was statistically associated with the magnitude of the viraemia at week 4 including: RNA level (*P* = 0.0052), RNA reduction (*P* = 0.0143), HCVcAg level (*P* = 0.0052) and HCVcAg reduction (*P* = 0.0135).

**Table 4:** Associations of HCV RNA and antigen measurements with SVR in TVR treatment.

**RNA-based indicator**	**Time point**	**SVR**	**Non-SVR**	***P*-value**
RNA level (log_10_ IU/ml), median	Baseline	5.83	6.29	**0.0456**
	Week 1	1.86	3.43	0.1111
	Week 2	1.38	2.36	0.2048
	Week 4	0.78	1.49	**0.0068**
RNA reduction (log_10_ IU/ml), median	Week 1	3.94	3.68	0.8756
	Week 2	4.24	4.68	0.5035
	Week 4	4.96	4.37	0.3117
RNA negativity, % (N)	Week 1	3.10	0.00	1.0000
	Week 2	12.50	0.00	1.0000
	Week 4	33.30	15.40	0.3356
**Antigen-based indicator**	**Time point**	**SVR**	**Non-SVR**	***P*-value**
Antigen level (log_10_ fmol/l), median	Baseline	3.43	3.61	0.1341
	Week 1	0.00	1.49	**0.0510**
	Week 2	0.00	0.71	0.0604
	Week 4	0.00	0.00	0.0766
Antigen reduction (log_10_ fmol/l), median	Week 1	2.64	2.60	0.9252
	Week 2	2.75	3.23	0.3914
	Week 4	3.07	3.19	0.8349
Antigen negativity, %	Week 1	53.10	0.00	0.2286
	Week 2	72.50	20.00	**0.0359**
	Week 4	81.10	61.50	0.1444

**Table 5:** Associations of HCV RNA and antigen measurements with SVR in BOC treatment.

**RNA-based indicator**	**Time point**	**SVR**	**Non-SVR**	***P*-value**
RNA level (log_10_ IU/ml), median	Baseline	6.12	6.24	0.3421
	Week 4	3.10	5.64	**0.0052**
RNA reduction (log_10_ IU/ml), median	Week 4	2.34	0.96	**0.0143**
RNA negativity, %	Week 4	7.10	0.00	1.0000
**Antigen-based indicator**	**Time point**	**SVR**	**Non-SVR**	***P*-value**
Antigen level (log_10_ fmol/l), median	Baseline	3.59	3.61	0.8545
	Week 4	0.89	3.11	**0.0052**
Antigen reduction (log_10_ fmol/l), median	Week 4	1.87	0.53	**0.0135**
Antigen negativity, n (N)	Week 4	32.10	0.00	0.0785

### Combination of predictive factors for SVR

For evaluation of the prognostic utility of the significant baseline and on-treatment SVR-predicting factors (presented in Tables 3–5), sensitivity, specificity, positive predictive value, negative predictive value and accuracy are summarised in Tables 6 and 7. The virological responses at weeks 8 and 12 were also included for comparison. ROC analysis was performed to calculate the optimal cut-off that represents the best compromise between sensitivity and specificity and to evaluate the respective predictive values of the continuous variables statistically associated with SVR.

**Table 6:** The most significant predictors of TVR therapy-induced SVR in CHC and their corresponding predictive values.

**On-treatment factor**	**Time point**	**Threshold**	***P*-value**	**AUC**	**SEN (%)**	**SPE (%)**	**PPV (%)**	**NPV (%)**	**ACC (%)**
RNA level	Baseline	≤5.78 log_10_ IU/ml	0.0141	0.685	48.31	90.91	97.7	17.9	53.0
RNA level	Week 4	≤1.26 log_10_ IU/ml	0.0078	0.726	76.67	69.23	94.5	30.0	75.7
Antigen level	Week 1	≤0.91 log_10_ fmol/l	0.0001	0.828	68.75	100	100	23.1	71.4
Antigen negativity	Week 2		0.0459		72.5	80	96.7	26.7	73.3
RNA negativity	Week 4		0.2256		31.5	85.7	84.0	16.0	38.3
RNA negativity	Week 12		0.0005		95.7	45.5	93.6	55.6	90.4
**Pre-treatment factor**	**Time point**	**Threshold**	***P*-value**	**AUC**	**SEN (%)**	**SPE (%)**	**PPV (%)**	**NPV (%)**	**ACC (%)**
rs12979860 CC	Baseline		0.0330		36.5	92.9	97.2	17.6	43.6
Age	Baseline	≤43 years old	<0.0001	0.714	48.96	100	100	22.2	54.5

**Table 7:** The most significant predictors of BOC therapy-induced SVR in CHC and their corresponding predictive values.

**On-treatment factor**	**Time point**	**Threshold**	***P*-value**	**AUC**	**SEN (%)**	**SPE (%)**	**PPV (%)**	**NPV (%)**	**ACC (%)**
RNA level	Week 4	≤3.23 log_10_ IU/ml	<0.0001	0.813	57.14	100	100	42.9	67.6
RNA reduction	Week 4	>1.25 log_10_ IU/ml	0.0002	0.804	74.07	85.71	95.2	46.2	73.5
Antigen level	Week 4	≤0.96 log_10_ fmol/l	<0.0001	0.812	53.57	100	100	40.9	64.7
Antigen reduction	Week 4	>1.45 log_10_ fmol/l	0.0002	0.807	59.26	100	100	38.9	64.7
RNA negativity	Week 8	Undetected RNA	0.1446		48.1	81.8	86.7	39.1	57.9
RNA negativity	Week 12	Undetected RNA	0.0013		87.1	70.0	90.0	63.6	82.9
**Pre-treatment factor**	**Time point**	**Threshold**	***P*-value**	**AUC**	**SEN (%)**	**SPE (%)**	**PPV (%)**	**NPV (%)**	**ACC (%)**
rs12979860 CC	Baseline		0.0410		32.3	100	100	34.4	50.0
Treatment-naïve	Baseline		0.0209		83.9	54.5	83.9	54.5	76.2

For both drugs, amongst all the parameters investigated, virological responses at week 12 provided the greatest degree of accuracy (90.6% and 82.9%, respectively). Failure to achieve these two virological responses was the strongest negative on-treatment predictor of inability to achieve an SVR (NPVs for TVR and BOC: 55.6% and 63.6%, respectively). At earlier time points, RNA negativity at week 4 in TVR and RNA negativity at week 8 in BOC provided lower predictive values (ACC) for TVR and BOC (38.3% and 57.9%, respectively).

For TVR therapy, at baseline, patient age (<43 years) possessed a higher accuracy in predicting SVR (54.5%) than pre-treatment viral load (53%) and *IFNL3* CC genotype (43.6%). Age is also the strongest positive predictor of SVR; 100% (43/43) of individuals <43 years of age achieved SVR. On treatment, after RNA negativity at week 12, both RNA levels at week 4 and HCVcAg negativity at week 2 gave comparable accuracies (75.7% and 73.3%, respectively). The best cut-off values for HCVcAg levels calculated at week 1 to maximise the prediction of SVR is 0.91 log_10_ fmol/l corresponding to 8.13 fmol/l. The optimal threshold for viral load at week 4 in SVR prediction is 1.26 log_10_ IU/ml equivalent to 18.2 IU/ml.

For BOC therapy, prior to starting treatment, *IFNL3* CC genotype is the strongest positive predictor of SVR (PPV: 100%), although the history of prior IFN-based therapy provided a greater accuracy (76.2% vs. 50.0%). On treatment, following RNA negativity at week 12, RNA reduction at week 4 gave the second highest accuracy (73.5%) with the optimal threshold of 1.25 log_10_ IU/ml. The other viraemia parameters at week 4 including RNA level, HCVcAg level and HCVcAg reduction gave lower accuracies than absolute RNA level; however, these three indicators are the strongest positive predictors of SVR (PPV: 100%). Measurement of HCVcAg level at week 4 gave the best cut-off at 0.96 log_10_ fmol/l for predicting SVR.

**Fig 3:**
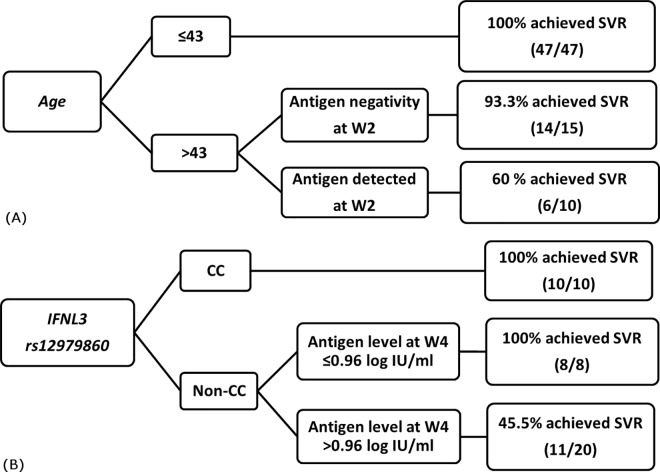
**Algorithm for identifying CHC patients receiving TVR (A) and BOC (B) with an increased likelihood to achieve SVR by combining pre-treatment and on-treatment factors.**

To generate an algorithm for prediction of the likelihood of SVR, significant predictors from the present study were selected for analysis. For TVR therapy, age, the strongest positive predictor at baseline, and antigen measurement at week 2 were combined to identify patients with a high probability of achieving SVR (Fig 3A). In the younger age group (<43 years), 100% achieved SVR. In the older age group (>43 years), patients with antigen negativity at week 2 achieved high SVR rates (93.3%). In the group with unfavourable characteristics in relation to both age (>43 years) and detectable antigen at week 2, the SVR rates were lower (60%). In BOC therapy, for a cost-effective approach targeting a general population, *IFNL3* and HCVcAg level at week 4 were combined for selection of individuals who are likely to achieve SVR (Fig 3B). Patients with *IFNL3* CC genotypes achieved a high SVR rate (100%) regardless of HCVcAg level at week 4. Furthermore, in the non-CC group, patients attained a high SVR rate (100%) if their HCVcAg level at week 4 decreased below 0.91 log_10_ fmol/l (equivalent to 8.12 fmol/l). In patients whose HCVcAg level was higher than the cut-off of 0.91 log_10_ fmol/l, the SVR rate was significantly lower (45.5%, *P* = 0.0291).

## Discussion

The cohort recruited in the ICORN study comprised 238 participants (170 patients receiving TVR and 68 patients receiving BOC). Due to the unavailability of samples for all patients at investigated time points for testing, 152 participants whose SVR evaluation was completed before September 2015 were included in the present study for measurement of HCVcAg and HCV RNA and correlation with treatment response. Of note, the overall SVR rates for the ICORN cohort (86.5% in TVR and 77.9% in BOC) are comparable to the rates reported in the present study (87.3% in TVR and 73.8% in BOC), which suggests that the patients in the present study are broadly representative of the whole ICORN cohort. The primary limitation of the present study relates to the small sample sizes, particularly in the BOC-treated cohort. The predictive value of quantitative HCVcAg in monitoring treatment response to DAA-based regimens requires confirmation in larger patient cohorts. Nevertheless, the information presented herein will potentially inform the study design for such projects.

The major aims of the present study were to evaluate the potential role for HCV core antigen in monitoring the response to triple therapies of TVR/BOC plus PEG-IFN/RBV and to determine the utility of HCVcAg testing in the era of DAAs. We report the outcome of treatment with PIs which suggests the usefulness of some baseline factors and antigen measurements in predicting favourable responses. It has been previously reported in clinical studies that various viral and patient characteristics could be predictive of responses to PI-based antiviral therapy [16–21]; however, the data have not been gathered into routine treatment guidelines due to the variations in the cohort characteristics and regimens employed in the investigations. In the present study, the factors predicting SVR exhibited strong positive predictive values; however, they yielded only a moderate capability of identifying individuals that would not achieve an SVR. In the analysis, we provide the algorithms for identifying CHC patients receiving PIs with an increased likelihood to achieve SVR by combining pre-treatment and on-treatment (antigen) factors in Fig 3. However, we acknowledge that the results were analysed with a relatively small cohort; therefore, the algorithms do not have implications for use in response-guided therapy. They are the illustration of the potential of antigen measurements in predicting SVR. To implement a practical strategy in real-life settings, studies with larger cohorts and further clinical and cost-benefit analyses will be required to establish firm recommendations for each nation. Consequently, the cost implications were not included in the present study. The high potency of the new DAA therapies attenuates the impact of baseline factors [22] but suggests the usefulness of antigen, an alternative marker in monitoring treatment response. IFN-free DAA therapy provides an opportunity to significantly simplify laboratory requirements and make the overall diagnostic package less expensive, particularly in resource-limited settings [23].

### Predictors of TVR treatment outcome

Among the baseline factors in our cohort for predicting SVR to TVR therapy, age had the highest association with outcome (*P* = 0.0098): TVR treatment for individuals younger than 43 years of age showed highest efficacy (100%). Host genetic polymorphisms at *IFNL3* rs12979860 were also significantly associated with treatment outcome (*P* = 0.0330) with 97.2% of individuals of *IFNL3* CC genotypes achieving SVR. Viral RNA titres at baseline in the SVR group (median: 5.83 log_10_ IU/ml, range: 3.39–7.08 log_10_ IU/ml) were lower than the non-SVR group (6.29 log_10_ IU/ml, range: 5.19–7.17 log_10_ IU/ml, *P =* 0.0456). On treatment, our results showed that antigen negativity at week 2 could provide the earliest indication that treatment would be successful. However, the utility of employing antigen negativity at week 2 requires investigation in larger cohorts receiving DAA therapy. In terms of identification of predictors at early stage, the dual factors of age and antigen at week 2 were included in the proposed model to identify patients predicted to have a higher likelihood of a successful therapeutic outcome.

### Predictors of BOC treatment outcome

Similar to TVR treatment, *IFNL3* genotype remained highly significantly associated with SVR in the BOC group: 100% of CC major homozygous individuals achieved SVR. Moreover, the failure of IFN-based therapy represented the strongest negative predictor at baseline of the failure to achieve an SVR. On treatment, week 4 viral parameters comprising RNA and HCVcAg measured by absolute or relative levels (reduction from baseline) were capable of predicting treatment response with BOC after the lead-in phase. Amongst these, measurement of HCVcAg at week 4 showed the highest predictive power for estimating SVR, with the further advantages of convenient data collection and cost-effectiveness. In the recommended treatment guidelines for BOC treatment, viral load testing at week 4 is not required for evaluation of virological response, and therefore HCVcAg testing could potentially replace expensive viral RNA quantification in monitoring treatment-induced virological responses at this time point. In the clinical setting, to target a general population, the model includes the dual factors of *IFNL3* genotype and antigen level at week 4 to determine patients who should initiate BOC therapy after the lead-in phase due to their favourable response.

The SOUND-C2 clinical trial examined faldaprevir and deleobuvir with and without ribavirin and demonstrated significantly higher SVR rates for rs12979860 CC homozygotes which highlighted the clinical relevance of *IFNL* genotyping in IFN-free DAA treatment regimens in chronic HCV treatment [24]. The rs12979860 SNP resides within the first intron of the newly described type III IFN, *IFN-λ4* (*IFNL4*) and the unfavourable rs12979860-T allele is in high linkage disequilibrium with a frameshift variant (rs368234815, *IFNL4-ΔG*) located within the first exon of *IFNL4* [25]. This *IFNL4-ΔG* frameshift establishes an open reading frame that allows IFNL4 expression [25]. Intrahepatic levels of interferon-stimulated genes (ISGs) are lower in rs12979860 CC homozygotes compared with non-CC individuals and similarly, the *IFNL4-ΔG* genotype has been associated with high intrahepatic ISG levels and treatment non-response [26]. This suggests that IFNL4 expression correlates with the transcriptional activation of ISGs, thus providing a molecular link between the host genotype and treatment-induced clearance of HCV [26, 27]. Therefore, mechanistically the clinical utility of *IFNL* genotypes are applicable to type I IFN-based and DAA treatment regimens.

For both PIs, whilst the baseline factors of treatment history, cirrhosis status and HCV genotype were not significantly associated with SVR in the present study, it was evident that there was a trend towards higher numbers of IFN-experienced, cirrhotic and HCV genotype 1a individuals in non-SVR groups than in SVR groups, which is in line with clinical trial data [17, 18, 28]. Conversely, the role of host factors such as IP-10 [29–32] and microRNAs [33] found to be capable of predicting SVR in dual PEG-IFN/RBV therapy were not associated with predicting the outcome in triple DAA therapy in the present study. The presence of natural occurring RAMs prior to treatment was shown to have no impact on treatment outcome, consistent with other studies [34, 35].

The association of *IFNL3* genotype and cirrhosis condition is also noteworthy in the present cohort. Several studies have reported the relationship of *IFNL3* genotypes and liver injury severity; however, the results showed discrepancies which could be due to study design, sample bias, viral genotypes or non-standard estimates of liver histology. In line with our study, some researchers have shown an association between the major homozygous *IFNL3* genotype (rs12979860 CC or rs8099917 TT) with severe portal inflammation [36], hepatic necroinflammation [37], fibrosis [38] and cirrhosis [39]. It has been suggested that *IFNL3* CC homozygotes, in whom treatment response rates are higher, but progression to cirrhosis may occur more rapidly would benefit the most in early access to treatment [39]. Conversely, an association between the unfavorable rs8099917 non-TT alleles (linked to the rs12979860 non-CC) and the risk of hepatocellular carcinoma has been reported [40] and the rs12979860 T allele frequency has also been reported to be more prevalence in patients with cirrhosis [41]. Presence of these alleles may affect the severity of liver fibrosis [42] and be associated with post-transplant fibrosis progression [43], or an increased risk for hepatocellular carcinoma in patients without SVR to dual therapy [44]. These contradictory findings highlight the need for further investigations to identify patients at greater risk of liver injury for more intensive monitoring.

### Clinical utility of HCVcAg

Despite being considered as an alternative to HCV RNA viral loads and supported by promising results in different studies of PEG-IFN/RBV treatment, HCVcAg measurements have not supplanted RNA measurements in clinical management [45]. In our study, we analysed the HCVcAg kinetics in the early stages of treatment and evaluated the predictive power of HCVcAg on SVR achievement. In TVR treatment, the antigen level at week 1 and antigen negativity at week 2 allowed distinction of the groups achieving different outcomes. Similar to the present study, Garbuglia and co-workers have reported the inability to detect HCVcAg one week after starting therapy is predictive of the likelihood to achieve SVR in TVR treatment [46]. Tamai and colleagues found no association between HCVcAg level and SVR of HCV specific genotype 1b and high viral loads [18], although in their previous study of interferon-based therapy, antigen levels within two weeks showed usefulness in predicting outcomes [10]. The variation of results could be due to different sample sizes and characteristics of the study cohorts. In BOC treatment, the earliest time point we could investigate was week 4 due to the numbers of clinical visits of patients at the earlier time points. The analysis showed promising results for the utility of antigen in monitoring virological response and predicting treatment outcomes. To our knowledge, we are the first group to evaluate the clinical utility of viral antigen measurements in BOC treatment outcome. The prognostic capability HCVcAg measurements at early stages should also be further substantiated in larger studies for newer DAAs.

The assay costs from the payer perspective of the HCVcAg and HCV viral load are €30 (~$34) and €65 (~$74) per test in Ireland, respectively. There are variations between laboratories or geographic regions for the cost of testing: the prices in the UK were $23.4 for antigen and $108 for viral load testing [47]; and in Egypt, antigen testing cost $19.8 while viral load testing was $141.5 [48]. In emerging economies, in spite of the lower labour costs, RNA viral load testing requires: a higher level of expertise for training and practice; greater capital investment for testing platforms; and longer processing time to perform the test, resulting in higher overall charges. In Vietnam, for example, the costs for commercial antigen and viral load testing in a medical laboratory are $32 and $79, respectively, which corresponds to the general price in other laboratories. HCVcAg assay requires considerably less hands-on time compared to the RNA assay: sample processing time for the HCVcAg testing is one hour, while that for viral load testing is 7 hours and 45 minutes. In real-life clinical practice, HCVcAg results could be issued potentially on the same day, or regularly one day after specimen collection; conversely, the turnaround time for viral load results is up to three days, largely due to the necessity to batch samples to optimise cost-effectiveness. Generally, the cost of HCV RNA testing is a multiple of the HCVcAg assay cost [23, 49]; therefore, the use of HCVcAg in place of HCV viral load testing will potentially help to decrease the cost of diagnosis, time to result and improve follow-up in certain settings. In terms of analytical performance, the antigen level was strongly correlated to viral load in CHC genotype 1 individuals; the reported data in larger cohorts indicated that there is no statistically significant difference in the viral RNA:HCVcAg correlation regarding different viral subtypes (1a vs. 1b) [50–53]. Hence, the strong correlation between viral load and HCVcAg at baseline facilitates the use of viral antigen monitoring to enable earlier commencement of therapy. With the introduction of new DAAs targeting HCV maturation and replication, HCVcAg could conceivably be superior to HCV RNA testing, as DAAs might already prevent virus formation and thereby better correlate with eventual therapy-induced viral clearance [45]. In the era of DAAs, the clinical significance of HCVcAg could be adjusted along with the sharp decrease of the levels of viraemia. On-treatment HCVcAg negativity would reflect the sustained efficacy of the novel drugs which are associated with very high SVR rates. It has been considered that IFN-free DAA therapy could simplify laboratory requirements and make the overall diagnostic package less expensive in low- and middle-income countries. HCVcAg testing could be employed to establish active infection, to initiate the requirement for treatment and then to evaluate aviraemia after cessation of DAA therapy [23]. Indeed, in a small scale study of liver transplant recipients undergoing treatment with an NS5B inhibitor (sofosbuvir), the interval time for HCVcAg loss was shown to be predictive of SVR outcome [54].

## Conclusions

The present study provides evidence for high positive predictive values of early quantitative HCVcAg, within two weeks in TVR-based regimen and four weeks in BOC-based regimen, in predicting SVR in chronic HCV genotype 1-infected patients receiving PI treatment. These results highlight the potential utility of early viral antigen kinetics in PI treatment and suggest that this could be a suitable replacement for viral load testing particularly in low and middle-income settings. The findings presented warrant further investigation to examine the role of antigen in monitoring treatment response in newer DAA regimens.

## Supporting Information

S1 Table Membership of the Irish Hepatitis C Outcomes Research Network (ICORN). (PDF)

## References

[1] Heim MH. 25 years of interferon-based treatment of chronic hepatitis C: an epoch coming to an end. Nature reviews Immunology. 2013;13(7):535–42. 10.1038/nri3463 . 23743475

[2] EASL. EASL Clinical Practice Guidelines: management of hepatitis C virus infection. Journal of hepatology. 2014;60(2):392–420. Epub 2013/12/18. 10.1016/j.jhep.2013.11.003 . 24331294

[3] Loggi E, Cursaro C, Scuteri A, Grandini E, Panno AM, Galli S, et al Patterns of HCV-RNA and HCV core antigen in the early monitoring of standard treatment for chronic hepatitis C. Journal of Clinical Virology. 2013;56(3):291–5. 10.1016/j.jcv.2012.11.012 23245628

[4] Morota K, Fujinami R, Kinukawa H, Machida T, Ohno K, Saegusa H, et al A new sensitive and automated chemiluminescent microparticle immunoassay for quantitative determination of hepatitis C virus core antigen. Journal of virological methods. 2009;157(1):8–14. Epub 2009/01/13. 10.1016/j.jviromet.2008.12.009 . 19135481

[5] Loggi E, Cursaro C, Scuteri A, Grandini E, Panno AM, Galli S, et al Patterns of HCV-RNA and HCV core antigen in the early monitoring of standard treatment for chronic hepatitis C. Journal of clinical virology: the official publication of the Pan American Society for Clinical Virology. 2013;56(3):207–11. Epub 2012/12/19. 10.1016/j.jcv.2012.11.012 . 23245628

[6] Veillon P, Payan C, Picchio G, Maniez-Montreuil M, Guntz P, Lunel F. Comparative evaluation of the total hepatitis C virus core antigen, branched-DNA, and amplicor monitor assays in determining viremia for patients with chronic hepatitis C during interferon plus ribavirin combination therapy. Journal of clinical microbiology. 2003;41(7):3212–20. Epub 2003/07/05. 10.1128/jcm.41.7.3212-3220.2003 12843066 PMC165326

[7] Vermehren J, Susser S, Berger A, Perner D, Peiffer KH, Allwinn R, et al Clinical utility of the ARCHITECT HCV Ag assay for early treatment monitoring in patients with chronic hepatitis C genotype 1 infection. Journal of clinical virology: the official publication of the Pan American Society for Clinical Virology. 2012;55(1):17–22. Epub 2012/06/16. 10.1016/j.jcv.2012.05.008 . 22698697

[8] Fujino T, Nakamuta M, Aoyagi Y, Fukuizumi K, Takemoto R, Yoshimoto T, et al Early decline of the HCV core antigen can predict SVR in patients with HCV treated by Pegylated interferon plus ribavirin combination therapy. Journal of digestive diseases. 2009;10(1):21–5. 10.1111/j.1751-2980.2008.00358.x . 19236543

[9] Wada Y, Tamai H, Uno A, Kawashima A, Shingaki N, Mori Y, et al Prediction of efficacy to pegylated interferon-alpha-2b plus ribavirin in patients with genotype 2 hepatitis C virus using viral response within 2 weeks. Hepatology research: the official journal of the Japan Society of Hepatology. 2014;44(2):179–86. 10.1111/hepr.12101 . 23531032

[10] Tamai H, Shingaki N, Shiraki T, Tukuda H, Mori Y, Moribata K, et al Prediction of sustained response to low-dose pegylated interferon alpha-2b plus ribavirin in patients with genotype 1b and high hepatitis C virus level using viral reduction within 2 weeks after therapy initiation. Hepatology research: the official journal of the Japan Society of Hepatology. 2011;41(12):1137–44. 10.1111/j.1872-034X.2011.00879.x . 21951330

[11] Tedder RS, Tuke P, Wallis N, Wright M, Nicholson L, Grant PR. Therapy-induced clearance of HCV core antigen from plasma predicts an end of treatment viral response. Journal of viral hepatitis. 2013;20(1):65–71. 10.1111/j.1365-2893.2012.01630.x . 23231086

[12] Collison M, Chin JL, Abu Shanab A, Mac Nicholas R, Segurado R, Coughlan S, et al Homozygosity for HLA group 2 alleles predicts treatment failure with interferon-alpha and ribavirin in chronic hepatitis C virus genotype 1 infection. Journal of interferon & cytokine research: the official journal of the International Society for Interferon and Cytokine Research. 2015;35(2):126–33. Epub 2014/09/23. 10.1089/jir.2014.0088 . 25237729

[13] Nguyen LT, Gray E, Dean J, Carr M, Connell J, De Gascun C, et al Baseline prevalence and emergence of protease inhibitor resistance mutations following treatment in chronic HCV genotype-1-infected individuals. Antiviral therapy. 2015;20(8):865–9. Epub 2015/04/30. 10.3851/imp2964 . 25920764

[14] Koberle V, Waidmann O, Kronenberger B, Andrei A, Susser S, Fuller C, et al Serum microRNA-122 kinetics in patients with chronic hepatitis C virus infection during antiviral therapy. Journal of viral hepatitis. 2013;20(8):530–5. 10.1111/jvh.12075 . 23808991

[15] EASL. EASL Clinical Practice Guidelines: management of hepatitis C virus infection. Journal of hepatology. 2014;60:392–420. 10.1016/j.jhep.2013.11.003 24331294

[16] Werner CR, Franz C, Egetemeyr DP, Beck R, Malek NP, Lauer UM, et al First-generation protease inhibitor-triple therapy: SVR 24, safety, and predictors of response in a large single center cohort. Virol J. 2015;12:37 10.1186/s12985-015-0261-0 25889921 PMC4355422

[17] Sterling RK, Kuo A, Rustgi VK, Sulkowski MS, Stewart TG, Fenkel JM, et al Virological outcomes and treatment algorithms utilisation in observational study of patients with chronic hepatitis C treated with boceprevir or telaprevir. Alimentary pharmacology & therapeutics. 2015;41(7):671–85. Epub 2015/01/30. 10.1111/apt.13095 ; PubMed Central PMCID: PMCPmc4529024. 25627020 PMC4529024

[18] Tamai H, Shimizu R, Shingaki N, Mori Y, Maeshima S, Nuta J, et al Prediction of Sustained Virological Response to Telaprevir-Based Triple Therapy Using Viral Response within 2 Weeks. Hepatitis research and treatment. 2014;2014:748935 10.1155/2014/748935 25328696 PMC4195394

[19] Shimada N, Toyoda H, Tsubota A, Ide T, Takaguchi K, Kato K, et al Baseline factors and very early viral response (week 1) for predicting sustained virological response in telaprevir-based triple combination therapy for Japanese genotype 1b chronic hepatitis C patients: a multicenter study. Journal of gastroenterology. 2014;49(11):1485–94. 10.1007/s00535-013-0918-7 . 24287582

[20] Yang CC, Tsai WL, Su WW, Huang CF, Cheng PN, Lo CC, et al Rapid Prediction of Treatment Futility of Boceprevir with Peginterferon-Ribavirin for Taiwanese Treatment Experienced Hepatitis C Virus Genotype 1-Infected Patients. PloS one. 2015;10(9):e0137852 10.1371/journal.pone.0137852 26368130 PMC4569190

[21] Cento V, Di Paolo D, Di Carlo D, Micheli V, Tontodonati M, De Leonardis F, et al Hepatitis C virus RNA levels at week-2 of telaprevir/boceprevir administration are predictive of virological outcome. Digestive and liver disease: official journal of the Italian Society of Gastroenterology and the Italian Association for the Study of the Liver. 2014;47(2):157–63. 10.1016/j.dld.2014.11.010 . 25544656

[22] EASL. EASL Recommendations on Treatment of Hepatitis C 2015. Journal of hepatology. 2015;63(1):199–236. Epub 2015/04/26. 10.1016/j.jhep.2015.03.025 . 25911336

[23] Cohn J, Roberts T, Amorosa V, Lemoine M, Hill A. Simplified diagnostic monitoring for hepatitis C, in the new era of direct-acting antiviral treatment. Current opinion in HIV and AIDS. 2015;10(5):369–73. Epub 2015/07/18. 10.1097/coh.0000000000000180 . 26185920

[24] Zeuzem S, Soriano V, Asselah T, Bronowicki JP, Lohse AW, Mullhaupt B, et al Faldaprevir and deleobuvir for HCV genotype 1 infection. N Engl J Med. 2013;369(7):630–9. Epub 2013/08/16. 10.1056/NEJMoa1213557 . 23944300

[25] Prokunina-Olsson L, Muchmore B, Tang W, Pfeiffer RM, Park H, Dickensheets H, et al A variant upstream of IFNL3 (IL28B) creating a new interferon gene IFNL4 is associated with impaired clearance of hepatitis C virus. Nature genetics. 2013;45(2):164–71. Epub 2013/01/08. 10.1038/ng.2521 ; PubMed Central PMCID: PMCPmc3793390. 23291588 PMC3793390

[26] Noureddin M, Rotman Y, Zhang F, Park H, Rehermann B, Thomas E, et al Hepatic expression levels of interferons and interferon-stimulated genes in patients with chronic hepatitis C: A phenotype-genotype correlation study. Genes and immunity. 2015;16(5):321–9. Epub 2015/05/29. 10.1038/gene.2015.11 . 26020282 PMC6015498

[27] Egli A, Santer DM, O'Shea D, Tyrrell DL, Houghton M. The impact of the interferon-lambda family on the innate and adaptive immune response to viral infections. Emerging microbes & infections. 2014;3(7):e51 Epub 2015/06/04. 10.1038/emi.2014.51 ; PubMed Central PMCID: PMCPmc4126180. 26038748 PMC4126180

[28] Poordad F, Bronowicki JP, Gordon SC, Zeuzem S, Jacobson IM, Sulkowski MS, et al Factors that predict response of patients with hepatitis C virus infection to boceprevir. Gastroenterology. 2012;143(3):608–18.e1-5. Epub 2012/05/26. 10.1053/j.gastro.2012.05.011 . 22626609

[29] Matsuura K, Watanabe T, Iijima S, Murakami S, Fujiwara K, Orito E, et al Serum interferon-gamma-inducible protein-10 concentrations and IL28B genotype associated with responses to pegylated interferon plus ribavirin with and without telaprevir for chronic hepatitis C. Hepatology research: the official journal of the Japan Society of Hepatology. 2014;44(12):1208–16. Epub 2014/01/01. 10.1111/hepr.12294 . 24372894

[30] Lagging M, Romero AI, Westin J, Norkrans G, Dhillon AP, Pawlotsky JM, et al IP-10 predicts viral response and therapeutic outcome in difficult-to-treat patients with HCV genotype 1 infection. Hepatology. 2006;44(6):1617–25. 10.1002/hep.21407 . 17133471

[31] Payer BA, Reiberger T, Aberle J, Ferenci P, Holzmann H, Rieger A, et al IL28B and interferon-gamma inducible protein 10 for prediction of rapid virologic response and sustained virologic response in HIV-HCV-coinfected patients. European journal of clinical investigation. 2012;42(6):599–606. 10.1111/j.1365-2362.2011.02623.x . 22117591

[32] Darling JM, Aerssens J, Fanning G, McHutchison JG, Goldstein DB, Thompson AJ, et al Quantitation of pretreatment serum interferon-gamma-inducible protein-10 improves the predictive value of an IL28B gene polymorphism for hepatitis C treatment response. Hepatology. 2011;53(1):14–22. 10.1002/hep.24056 21254158 PMC3083026

[33] Su TH, Liu CH, Liu CJ, Chen CL, Ting TT, Tseng TC, et al Serum microRNA-122 level correlates with virologic responses to pegylated interferon therapy in chronic hepatitis C. Proceedings of the National Academy of Sciences of the United States of America. 2013;110(19):7844–9. 10.1073/pnas.1306138110 23613588 PMC3651447

[34] Larrat S, Vallet S, David-Tchouda S, Caporossi A, Margier J, Ramiere C, et al Naturally Occurring Resistance-Associated Variants of Hepatitis C Virus Protease Inhibitors in Poor Responders to Pegylated Interferon-Ribavirin. 2015;53(7):2195–202. 10.1128/jcm.03633-14 . 25926499 PMC4473242

[35] Howe JA, Long J, Black S, Chase R, McMonagle P, Curry S, et al Clinical Implications of Detectable Baseline Hepatitis C Virus-Genotype 1 NS3/4A-Protease Variants on the Efficacy of Boceprevir Combined With Peginterferon/Ribavirin. Open forum infectious diseases. 2014;1(2):ofu078 Epub 2015/03/04. 10.1093/ofid/ofu078 ; PubMed Central PMCID: PMCPmc4281806. 25734146 PMC4281806

[36] D'Ambrosio R, Aghemo A, De Francesco R, Rumi MG, Galmozzi E, De Nicola S, et al The association of IL28B genotype with the histological features of chronic hepatitis C is HCV genotype dependent. International journal of molecular sciences. 2014;15(5):7213–24. 10.3390/ijms15057213 24776764 PMC4057668

[37] Noureddin M, Wright EC, Alter HJ, Clark S, Thomas E, Chen R, et al Association of IL28B genotype with fibrosis progression and clinical outcomes in patients with chronic hepatitis C: a longitudinal analysis. Hepatology. 2013;58(5):1548–57. 10.1002/hep.26506 23703931 PMC3758382

[38] Eslam M, Hashem AM, Leung R, Romero-Gomez M, Berg T, Dore GJ, et al Interferon-lambda rs12979860 genotype and liver fibrosis in viral and non-viral chronic liver disease. Nature communications. 2015;6:6422 10.1038/ncomms7422 25740255 PMC4366528

[39] Barreiro P, Pineda JA, Rallon N, Naggie S, Martin-Carbonero L, Neukam K, et al Influence of interleukin-28B single-nucleotide polymorphisms on progression to liver cirrhosis in human immunodeficiency virus-hepatitis C virus-coinfected patients receiving antiretroviral therapy. The Journal of infectious diseases. 2011;203(11):1629–36. Epub 2011/05/20. 10.1093/infdis/jir113 . 21592993

[40] Asahina Y, Tsuchiya K, Nishimura T, Muraoka M, Suzuki Y, Tamaki N, et al Genetic variation near interleukin 28B and the risk of hepatocellular carcinoma in patients with chronic hepatitis C. Journal of gastroenterology. 2014;49(7):1152–62. 10.1007/s00535-013-0858-2 . 23860735

[41] Fabris C, Falleti E, Cussigh A, Bitetto D, Fontanini E, Bignulin S, et al IL-28B rs12979860 C/T allele distribution in patients with liver cirrhosis: role in the course of chronic viral hepatitis and the development of HCC. Journal of hepatology. 2011;54(4):716–22. Epub 2010/12/15. 10.1016/j.jhep.2010.07.019 . 21146242

[42] Falleti E, Bitetto D, Fabris C, Cussigh A, Fornasiere E, Cmet S, et al Role of interleukin 28B rs12979860 C/T polymorphism on the histological outcome of chronic hepatitis C: relationship with gender and viral genotype. Journal of clinical immunology. 2011;31(5):891–9. Epub 2011/06/08. 10.1007/s10875-011-9547-1 . 21647799

[43] Eurich D, Boas-Knoop S, Bahra M, Neuhaus R, Somasundaram R, Neuhaus P, et al Role of IL28B polymorphism in the development of hepatitis C virus-induced hepatocellular carcinoma, graft fibrosis, and posttransplant antiviral therapy. Transplantation. 2012;93(6):644–9. Epub 2012/03/14. 10.1097/TP.0b013e318244f774 . 22411462

[44] Chang KC, Tseng PL, Wu YY, Hung HC, Huang CM, Lu SN, et al A polymorphism in interferon L3 is an independent risk factor for development of hepatocellular carcinoma after treatment of hepatitis C virus infection. Clinical gastroenterology and hepatology: the official clinical practice journal of the American Gastroenterological Association. 2015;13(5):1017–24. 10.1016/j.cgh.2014.10.035 . 25460552

[45] Tillmann HL. Hepatitis C virus core antigen testing: role in diagnosis, disease monitoring and treatment. World journal of gastroenterology: WJG. 2014;20(22):6701–6. 10.3748/wjg.v20.i22.6701 24944462 PMC4051911

[46] Garbuglia AR, Lionetti R, Lapa D, Taibi C, Visco-Comandini U, Montalbano M, et al The clinical significance of HCV core antigen detection during Telaprevir/Peg-Interferon/Ribavirin therapy in patients with HCV 1 genotype infection. Journal of clinical virology: the official publication of the Pan American Society for Clinical Virology. 2015;69:68–73. 10.1016/j.jcv.2015.06.002 . 26209382

[47] Cresswell FV, Fisher M, Hughes DJ, Shaw SG, Homer G, Hassan-Ibrahim MO. Hepatitis C core antigen testing: a reliable, quick, and potentially cost-effective alternative to hepatitis C polymerase chain reaction in diagnosing acute hepatitis C virus infection. Clinical infectious diseases: an official publication of the Infectious Diseases Society of America. 2015;60(2):263–6. Epub 2014/10/11. 10.1093/cid/ciu782 . 25301216

[48] Kamal SM, Kassim S, El Gohary E, Fouad A, Nabegh L, Hafez T, et al The accuracy and cost-effectiveness of hepatitis C core antigen assay in the monitoring of anti-viral therapy in patients with chronic hepatitis C genotype 4. Alimentary pharmacology & therapeutics. 2015;42(3):307–18. Epub 2015/05/29. 10.1111/apt.13261 . 26018116

[49] Kadkhoda K, Smart G. HCV antigen testing for the diagnosis of hepatitis C infection: a cost-efficient algorithm. Clinical laboratory. 2014;60(4):677–80. Epub 2014/05/02. . 24779304 10.7754/clin.lab.2013.130634

[50] Nguyen LT, Dunford L, Freitas I, Holder P, Nguyen LA, O'Gorman J, et al Hepatitis C Virus Core Mutations Associated with Genotype 3a False Negative Antigen Serology. Journal of clinical microbiology. 2015;53(8):2697–700. Epub 2015/05/23. 10.1128/jcm.01062-15 . 25994168 PMC4508445

[51] Chevaliez S, Soulier A, Poiteau L, Bouvier-Alias M, Pawlotsky JM. Clinical utility of hepatitis C virus core antigen quantification in patients with chronic hepatitis C. Journal of clinical virology: the official publication of the Pan American Society for Clinical Virology. 2014;61(1):145–8. 10.1016/j.jcv.2014.05.014 . 24973282

[52] Medici MC, Furlini G, Rodella A, Fuertes A, Monachetti A, Calderaro A, et al Hepatitis C virus core antigen: analytical performances, correlation with viremia and potential applications of a quantitative, automated immunoassay. Journal of clinical virology: the official publication of the Pan American Society for Clinical Virology. 2011;51(4):264–9. 10.1016/j.jcv.2011.05.003 . 21621454

[53] Ottiger C, Gygli N, Huber AR. Detection limit of architect hepatitis C core antigen assay in correlation with HCV RNA, and renewed confirmation algorithm for reactive anti-HCV samples. Journal of clinical virology: the official publication of the Pan American Society for Clinical Virology. 2013;58(3):535–40. 10.1016/j.jcv.2013.08.028 . 24041472

[54] Pischke S, Polywka S, Proske VM, Lang M, Jordan S, Nashan B, et al Course of hepatitis C virus (HCV) RNA and HCV core antigen testing are predictors for reaching sustained virologic response in liver transplant recipients undergoing sofosbuvir treatment in a real-life setting. Transplant infectious disease: an official journal of the Transplantation Society. 2015;18(1):141–5. Epub 2015/10/21. 10.1111/tid.12475 . 26485543

