# Supplementary material for: The Role of Hepatitis C Virus Core Antigen Testing in the Era of Direct Acting Antiviral Therapies: What We Can Learn from the Protease Inhibitors
Source: PLoS One. 2016 Oct 6;11(10):e0163900. doi: 10.1371/journal.pone.0163900 (PMC5053597; doi:10.1371/journal.pone.0163900)
Supplement: S1 Table — (PDF) [file pone.0163900.s001.pdf]

SUPPORTING INFORMATION

RESEARCH ARTICLE

Title: The Role of Hepatitis C Virus Core Antigen Testing in the Era of Direct Acting Antiviral Therapies: What We Can Learn from the Protease Inhibitors

S1 Table. Membership of Irish Hepatitis C Outcomes Research Network (ICORN)

No.	Name	Hospital/Organisation/Institute
1	Suzanne Norris *(Principal investigator)*	Department of Hepatology, St. James's Hospital, Dublin 8
2	Michael Barry	Department of Pharmacology & Therapeutics, St James's Hospital, Dublin 8
3	Colm Bergin	St James's Hospital, Dublin 8
4	Mary Cannon	Beaumont Hospital, Dublin
5	Jeff Connell	UCD National Virus Reference Laboratory, Belfield, Dublin 4
6	Suzie Coughlan	UCD National Virus Reference Laboratory, Belfield, Dublin 4
7	Miriam Coghlan	Pharmacy Department, Hospital 7, St. James's Hospital, Dublin 8
8	Garry Courtney	St. Luke’s Hospital, Kilkenny
9	Grainne Cousins	Royal College of Surgeons in Ireland, Dublin 2
10	Orla Crosbie	University Hospital Cork, Cork
11	Cillian De Gascun	UCD National Virus Reference Laboratory, Belfield, Dublin 4
12	Liam Fanning	Department of Medicine, Cork University Hospital, Cork
13	Kathryn Feeley	St Vincent’s University Hospital, Elm Park, Dublin 4
14	Eoin Feeney	St. Vincent's University Hospital, Elm Park, Dublin 4
15	Catherine Fleming	Galway University Hospital, Galway
16	Clair Gardiner	School of Biochemistry and Immunology, TCD
17	Emma Gray	National Centre for Pharmacoeconomics in Ireland, St. James's Hospital, Dublin 8
18	Sheila Heffernan	HSE National Drug Treatment Centre, Dublin 2
19	John Hegarty	St Vincent’s Hospital, Dublin
20	Diarmaid Houlihan	St Vincent’s Hospital, Dublin
21	Helena Irish	St. James's Hospital, Dublin 8
22	Shay Keating	National Drug Treatment Centre

23	Jennifer Ann Kieran	Department of Pharmacology & Therapeutics, St James's Hospital, Dublin 8
24	Barry Kelleher	Mater Misericordiae University Hospital, Dublin 7
25	John Lambert	Mater, Rotunda & UCD Dublin
26	John Lee	University Hospital Galway, Galway
27	Paddy Mallon	UCD School of Medicine and Medical Science, UCD, Belfield, Dublin 4
28	Samuel McConkey	Royal College of Surgeons in Ireland, Dublin 2
29	Aiden McCormick	St Vincent’s Hospital, Dublin
30	Susan McKiernan	St. James's Hospital, Dublin 8
31	John Moloney	HSE, Co Dublin
32	Busi Mooka	University of Limerick
33	Frank Murray	Beaumont Hospital, Dublin
34	Ursula Norton	Wheatfield Place of Detention, Dublin 22
35	Mairead O'Connor	Pharmacy Department, Mater Misericordiae University Hospital, Dublin 7
36	Cliona O'Farrelly	Trinity BioSciences Institute, Trinity College, Dublin 2
37	Aisling O'Leary	RCSI School of Pharmacy, Royal College of Surgeons in Ireland, Dublin 2
38	Brian O'Mahony	Irish Haemophilia Society Ltd, Dublin 8
39	Nicola Perry	Community Response, Carman’s Hall, Dublin 8
40	Mark Robinson	Trinity Biomedical Sciences Institute, Trinity College, Dublin 2
41	Nigel John Stevenson	Trinity Biomedical Sciences Institute, Trinity College Dublin, Dublin 2
42	Stephen Stewart	Mater Misericordiae University Hospital, Dublin 7
43	Michele Tait	HSE, Mill Lane, Palmerstown, Dublin 20
44	Lelia Thornton	Health Protection Surveillance Centre, Dublin 1
45	Cathal Walsh	University of Limerick
46	Caroline Walsh	Liver Unit in the Mater Misericordiae University Hospital, Dublin 7
